# Pragmatic considerations for fostering reproducible research in artificial intelligence

**DOI:** 10.1038/s41746-019-0120-2

**Published:** 2019-05-22

**Authors:** Rickey E. Carter, Zachi I. Attia, Francisco Lopez-Jimenez, Paul A. Friedman

**Affiliations:** 1 Department of Health Sciences Research, Mayo Clinic, Jacksonville, FL USA; 2 Department of Cardiovascular Disease, Mayo Clinic, Rochester, MN USA

**Keywords:** Medical research, Intellectual-property rights

## Abstract

Artificial intelligence and deep learning methods hold great promise in the medical sciences in areas such as enhanced tumor identification from radiographic images, and natural language processing to extract complex information from electronic health records. Scientific review of AI algorithms has involved reproducibility, in which investigators share protocols, raw data, and programming codes. Within the realm of medicine, reproducibility introduces important challenges, including risk to patient privacy, challenges in reproducing results, and questions regarding ownership and financial value of large medical datasets. Scientific review, however, mandates some form of resolution of these inherent conflicts. We propose several approaches to permit scientific review while maintaining patient privacy and data confidentiality.

## Introduction

Artificial intelligence (AI) utilizing deep learning methods holds great promise in the medical sciences.^1–3^ Rapid exploration of these machine learning techniques is leading to pioneering new insights ranging from natural language processing of electronic health records^4^ to high-volume image processing that has classification performance on par with highly trained humans.^5^ There is promise that the development of AI-based solutions will help guide the future of precision medicine.^6^ The promise is large,^7,8^ but certain aspects of applied AI research are raising concerns.^9,10^ One critical concern that warrants attention is how to promote sound scientific practices, including replication of findings implementing reproducible research and data-sharing practices in the era of AI.

Replication, the process of independently repeating an experiment and arriving at the same conclusion, is one of the hallmarks of the scientific process. While the conclusion may be the same, the underlying data supporting the conclusion may be different (i.e., not replicated) due to random variation or other causes such as slightly changed experimental conditions. Complementary to this concept is the process for which evidence for the inadequacy of the approach is detailed, an approach that aligns well with the falsification philosophy pioneered by Karl Popper.^11^ While replication and falsification are desirable, they are not always practical or advantageous in all settings.^12^ As an alternative, reproducible research has been introduced as a pragmatic approach in which investigators create research transparency by sharing the protocols, raw data, programming codes, and insights used to generate the final summary.^12^ Recently, the concept of reproducibility has been extended through a call for the dissemination of the full ‘scientific recipe'.^13^ While this is a laudable goal and one that could be easily achieved for many small basic and clinical studies using routine methodology, the incorporation of AI into research raises practical challenges to reproducible research practices. Nonetheless, a robust means of ensuring reproducible AI research is urgently needed. The objective of this perspective is to share some concepts to help facilitate reproducible AI research focused on data-sharing challenges and algorithm development.

## Start with raw data, but what is the raw data?

The practice of reproducible research in the context of AI, in principle, should encompass the raw data generation process along with all of the programming syntax that transforms the data into the numerical summaries that accompany a scientific report. In this way, every step in the process is clearly documented and subjected to inspection. Raw data traditionally consisted of information downloaded directly from instrumentation (e.g., electrocardiogram) or obtained directly from a participant through interview. AI often utilizes non-traditional data sources, such as clinical notes and medical images, including photographs.^14^ Such data sources may be linked with protected health information to the point where sharing in a de-identifiable manner is not feasibly possible. This is a departure from research in which de-identification might be as simple as removing a subject ID from a file. How would one, for example, share de-identified patient photographs used to train an algorithm? A black censorship bar irrevocably destroys the data for training purposes. The problem is complex. A human face can be reconstructed from a head magnetic resonance imaging dataset.^15^ Clinical notes that describe a family history of a rare disease would need to be rewritten to de-identify them. Hence, while reproducible research may emphasize the need for the raw data, many healthcare-based datasets used for AI research are not always feasibly releasable. For these reasons and others, such as scientific competiveness or intellectual property considerations, one encounters statements such as “The data, analytic methods, and study materials will not be made available to other researchers for purposes of reproducing the results or replicating the procedure”^16^ in the literature.

## Who pays for the cost of data collection, and who can determine if the data can be shared?

Large volumes of well-curated data are essential for training and validating AI algorithms. Hospitals, for example, have invested significant resources in the acquisition and support of electronic medical records, funding specialist salaries, and acquiring and maintaining expensive equipment to collect the datasets that are used to develop AI systems. Even in instances where public funding was used to support the development of an AI system, the data used in the model training may have been collected and curated over many years without this support. Should organizations be encouraged or required to share these data with the public in exchange for publication? This is a decision that extends beyond that of the authors’ and journal’s beliefs. Furthermore, sharing of health-care data raises questions about the role of the patient in this decision-making process.^17^ In some states within the United States, explicit authorization by the patient is required to utilize medical records for research.^18^ Similarly, the European Union’s recently enacted general data protection regulation is raising questions about data access, use, and sharing policies across the world.^19–21^ Thus, while public sharing of data is highly desirable as it is a means to support important medical advances, it cannot be universally recommended or required.

## Is reproducibility as simple as just running the code?

Ideally, with the raw data and programming code, reproducing a study’s reported summaries should be as simple as “push the button”. For statistical models such as least-squares regression, results can be routinely reproduced. AI, however, is a careful balance of a rapidly evolving set of hardware and software. The data utilized often require significant pre-processing to move it from the raw data state to an analysis-ready state. Even with the data and algorithms available, specialized workstations utilizing the exact software and hardware (e.g., graphics processing units) may be required to reproduce the results. This is not a trivial expectation given the cost and availability of specialized high performance computing infrastructure. More troubling, however, is that having the analytical data, programming code, and the entire necessary computing environment may not be sufficient to exactly reproduce the estimation process. There is knowledge within the development community that small computational variances occur over a wide range of software and versions when using a graphics processing unit for high-performance computing (https://github.com/tensorflow/tensorflow/issues/2732). These variances are not eliminated by setting a starting number for random number generation as they are when software is not massively parallelized (i.e., run on a central processing unit). Simply put, it may not be possible to identically regenerate the model as it is with models that have closed-form solutions (i.e., “equations”). This realization is such that the raw data and programming code are not enough to overcome the reproducibility crisis facing AI research.^9^ It should be noted, however, that once the model has been estimated, deterministic and reproducible results are obtained from the final fitted model.

## Where do we go next?

This is one of the most important questions in AI research. If the goal is to produce generalizable knowledge and support transparency in the research process, then there is a compelling need to find a way to share data and programs to ensure reproducibility while protecting patient privacy, financial investments, and intellectual property. Publically available data and programs provide excellent learning opportunities for scientists seeking to explore and extend computational techniques, which is laudable but not aligned with reproducible research. Prior to formulating recommendations, it is important to understand why we need reproducible research. It is not to altruistically advance science through sharing data and programs; it is to enhance the scientific rigor of research and to ensure that claims are valid.^22^

As noted above, sharing data and programming code used for AI algorithm development and testing is inherently more complex than traditional research. One approach for transparency in the AI research process that overcomes these challenges is a virtual review of the actual programming environment used for model training—the equivalent of a regulatory inspection. Here, the “inspection” could be conducted using real-time screen captures and narrated code explanation. For example, the programming code could be discussed in more detail than could be explained in written form by the analyst while summarizing and executing key code on the screen. This would allow for illustrations of the data objects used in modeling while showing the written programming code and how the code performs in the actual training environment. This record could be easily shared broadly and archived using standard video sharing platforms. Its availability would enhance the written summaries that would be included in manuscripts, and possibly help address the technical limitations of scientists trained in traditional statistical approaches and possessing limited exposure to machine learning methods. A key strength of this approach is reducing or eliminating the technical and regulatory barriers associated with public release.

Another more technically advanced solution would be to create a protected computing environment (“data enclave” or “sandbox”) that reviewers could log in to and explore. The data and modeling could be made available in this environment in a read-only, non-exportable fashion. This extends the video environment to one in which the reviewer can actually “push the button”. A downside of this approach is that it would be costly to maintain in perpetuity and assumes the inspector has adequate training and understanding to operate the controls. Furthermore, if the goal is to validate the training of the model, which ultimately appears to be the interest when reproducible research is described, significant time and expense for the computation should be expected. Even with the fastest graphics processing units available today, model estimation can extend over days, weeks, or months. Once the model has been estimated, the use of the model for cases such as prediction of results in the test data is much more time efficient. However, the scientific importance of validating this step might be of lower importance and not sufficient to warrant building and maintaining the computing infrastructure.

An extension of the data enclave approach would be to distribute, through an appropriate license agreement, an application that houses the trained algorithm and necessary support code to run the algorithm. In this way, the intellectual property associated with the algorithm can be managed and users would be able to examine the performance on his or her own data. The need for data transfer would thus be avoided. Testing the algorithm on new populations would add important information on the generalizability of estimates obtained from the AI algorithm. It is worth noting that if an AI algorithm fails to generalize it may be a result of irreproducible research (e.g., spurious associations learned by the AI algorithm) or it could be related to patient heterogeneity; both issues are worthy of exploration. A limitation of this approach is that not all academic investigators or institutions are well positioned to develop and distribute software solutions. The distribution and maintenance of licensed software would require a structured business plan in order to be successful, and such a business model may evolve well after the initial modeling work has been completed.

The most technologically advanced solution would be to have data and the entire computing infrastructure made available to interested parties in the context of legally binding agreements to ensure data security, patient confidentiality, and appropriate ownership of intellectual property. Subject matter expertise would also be vital in these contractual agreements. Consistent with any research involving human subjects, the absence of a subject matter expert could result in a significant risk for spurious statistical associations, false conclusions, and harm. Moreover, for expert medical networks to be useful, someone must take responsibility and ownership of those. There should be alignment between this responsibility and potential indemnification, and financial reward for institutions involved in their development. This would require a high-level partnership of organizations, and, as such, may be well beyond the intentions of reproducible science.

Of the possible solutions for reproducibility for AI, the mixed media approach of combining written technical summaries in the form of a manuscript with runtime video capture showing the compilation and utilization of the computing environment is by far the most pragmatic, particularly for supporting peer review of manuscripts and early communication of the AI algorithm’s results. This type of approach mirrors how computer programming is taught in both in-person and online courses, so it will be readily understood by the target audience.

## Conclusions

In the end, we need to take concrete steps toward mitigating irreproducible research through increased transparency. This is particularly relevant as cardiology transitions from risk estimation using classical tools such as the Framingham Risk Score—tools that make it explicitly clear what drives the model predictions and are easily validated in new populations—to digital signatures that are derived from advanced data networks of a scale and complexity not conducive to hand tabulation. There are many barriers to reproducible AI research, including the speed at which technology is moving. We need to challenge ourselves to overcome these barriers with innovative approaches. However, we must also recognize that research with human-derived measures of performance also faces challenges with reproducibility, and that many of the recommendations made herein may exceed the reproducibility expectations for such studies. Ultimately, there must be some degree of trust within the scientific community that appropriate methods were utilized, and reproducible research practices can help build this trust. This need for trust is ironically one of the most important human elements in AI research.
